# A key role for STIM1 in store operated calcium channel activation in airway smooth muscle

**DOI:** 10.1186/1465-9921-7-119

**Published:** 2006-09-20

**Authors:** Samantha E Peel, Bo Liu, Ian P Hall

**Affiliations:** 1 Division of Therapeutics and Molecular Medicine, University Hospital, Queens Medical Centre, Nottingham, UK

## Abstract

**Background:**

Control of cytosolic calcium plays a key role in airway myocyte function. Changes in intracellular Ca^2+ ^stores can modulate contractile responses, modulate proliferation and regulate synthetic activity. Influx of Ca^2+ ^in non excitable smooth muscle is believed to be predominantly through store operated channels (SOC) or receptor operated channels (ROC). Whereas agonists can activate both SOC and ROC in a range of smooth muscle types, the specific trigger for SOC activation is depletion of the sarcoplasmic reticulum Ca^2+ ^stores. The mechanism underlying SOC activation following depletion of intracellular Ca^2+ ^stores in smooth muscle has not been identified.

**Methods:**

To investigate the roles of the STIM homologues in SOC activation in airway myocytes, specific siRNA sequences were utilised to target and selectively suppress both STIM1 and STIM2. Quantitative real time PCR was employed to assess the efficiency and the specificity of the siRNA mediated knockdown of mRNA. Activation of SOC was investigated by both whole cell patch clamp electrophysiology and a fluorescence based calcium assay.

**Results:**

Transfection of 20 nM siRNA specific for STIM1 or 2 resulted in robust decreases (>70%) of the relevant mRNA. siRNA targeted at STIM1 resulted in a reduction of SOC associated Ca^2+ ^influx in response to store depletion by cyclopiazonic acid (60%) or histamine but not bradykinin. siRNA to STIM2 had no effect on these responses. In addition STIM1 suppression resulted in a more or less complete abrogation of SOC associated inward currents assessed by whole cell patch clamp.

**Conclusion:**

Here we show that STIM1 acts as a key signal for SOC activation following intracellular Ca^2+ ^store depletion or following agonist stimulation with histamine in human airway myocytes. These are the first data demonstrating a role for STIM1 in a physiologically relevant, non-transformed endogenous expression cell model.

## Background

Control of intracellular calcium is critical to regulation of smooth muscle function in many tissues. The relative contribution of SOC to the control of intracellular Ca^2+ ^varies between different types of smooth muscle, with SOC being particularly prominent in airway myocytes. The contractile/relaxant state of the airway myocyte is a key determinant of airway calibre thus contributing to bronchoconstriction in diseases such as asthma. Previous studies have demonstrated that the contractile response of airway myocytes is dependent initially upon release of intracellular Ca^2+ ^from the sarcoplasmic reticulum (reviewed in [1]) but that sustained contraction is dependent upon influx from extracellular sources. Two mechanisms have been proposed to account for this influx in airway myocytes involving either activation of SOC or ROC. In contrast to vascular smooth muscle, L type voltage dependent calcium channels (VDCCs) appear to play a negligible role in control of Ca^2+ ^entry [2]. In previous work we have demonstrated the expression of a number of TRP homologues including TRPC1, 3, 4 and 6 in cultured human airway myocytes and lung tissue and have suggested that TRPC6 may play an important role (probably together with other TRPC homologues including TRPC3 [3]) in contributing to agonist induced ROC activity [4].

SOC activation in many cell types including smooth muscle is known to involve depletion of the intracellular sarcoplasmic reticulum Ca^2+ ^stores. Contractile agonists such as acetylcholine, histamine and bradykinin may vary in their ability to differentially activate ROC or SOC although all agonists are known to induce activation of phospholipase C with consequent IP_3 _mediated Ca^2+ ^release from the intracellular stores. The mechanism underlying signalling for subsequent Ca^2+ ^influx in response to store depletion, and hence refilling of the sarcoplasmic reticulum Ca^2+ ^stores, remains unknown. In the current study we have set out to define the signals for SOC activation in human airway myocytes following both store depletion and agonist activation by spasmogens.

Using RNA interference techniques STIM (stromal interaction molecule) 1 has been shown to play a role in SOC induced calcium entry in Drosophilia S2 cells, Jurkat T cells [5] and Hela cells [6] with the latter study also implicating a role for STIM2. In particular, STIM1 appears to be a major activator of calcium release activated calcium channels (I_CRAC_) in T lymphocytes via a mechanism which has been proposed to involve translocation of STIM1 from endoplasmic reticulum like sites to the cell membrane [7]. We therefore hypothesised that homologues of STIM may play a role in SOC activation in smooth muscle. To address this hypothesis we used specific siRNA sequences to suppress both STIM1 and STIM2.

## Methods

### Cells

Human bronchial tissue was obtained from patients without a history of asthma. Human airway smooth muscle (HASM) cells were isolated and cultured as previously described [8]. Ethical approval for these studies was obtained from the Nottingham local ethical research committee. All subjects from whom tissue was obtained gave written consent. Primary human bronchial epithelial cells were obtained from Cambrex Bioscience (MD, USA) and grown in accordance with suppliers protocols. Cells at passage 4 were differentiated at an air-liquid interface on polyester tissue culture inserts (Corning, Costar) as described in a previous published method [9].

### Transfection of siRNAs

siRNAs, including the scrambled siRNA control were purchased from Ambion (Huntingdon, Cambridge, UK). STIM1 siRNA (AAGGGAAGACCTCAATTACCA) was pre-designed from Ambion, STIM2 siRNA (AACTGAGAAGCAGTTGGTCTG) designed by Roos and colleagues [5]. Cells were transfected with siRNA (1–50 nM) in serum free medium over a period of 6 h, the medium was then aspirated and replaced with serum containing medium for a further period of 42 h. The transfection reagent used was Lipofectamine 2000 (Invitrogen, Paisley, UK) at a final concentration of 2 μl/ml.

### Total RNA extraction and reverse transcriptase PCR

Total RNA was isolated from pelleted cells using the RNeasy mini kit (Qiagen, West Sussex, UK) as per manufacturers' instructions. To examine for STIM1 and STIM2 expression, RNA was reverse-transcribed using Superscript II reverse transcriptase (Invitrogen) and random hexamers (Invitrogen). PCR was performed using specific primers against STIM1 (Forward; AGGCAGTCCGTAACATCCAC, Reverse; CTTCAGTCCGTAACATCCAC) and STIM2 (Forward; TCCCTGCATGTCACTGAGTC, Reverse; GGGAAGTGTCGTTCCTTTGA). Cycling was performed 35 times; 94°C, followed by 55°C (annealing temperature), then 72°C (all for 90 seconds) followed by 10 mins at 72°C. PCR products were visualized by ethidium bromide staining and confirmed by direct sequencing.

### Real-Time PCR (Taqman)

siRNA targeted mRNA knockdown was measured using real time, quantitative PCR (Taqman). Gene specific primers and probes against STIM1 and STIM2 were designed using Primer Express™ software (Applied Biosystems, Foster City, CA) and using 18s RNA as the reference gene (Applied Biosystems). All probes were MGB probes, labeled with a 5'-reporter dye FAM and a non fluorescent quencher. Each sample was run in duplicate and mRNA knockdown was measured from mRNA obtained from 3 separate experiments. The relative expression of the target gene was calculated using the comparative method (2^-ΔΔCt^) [10].

Primer and probe sequences:

STIM-1 forward primer: AAGGCTCTGGATACAGTGCTCTTT

reverse primer: AGCATGAAGTCCTTGAGGTGATTAT

probe: CTCCTCTCTTGACTCGC

STIM-2 forward primer: ACGACACTTCCCAGGATAGCA

reverse primer: GACTCCGGTCACTGATTTTCAAC

probe: TGCACGAACCTTCATT

### Measurement of [Ca2+]_i_

HASMs (passage 4–5) were plated in black walled, clear bottom 96 well plates and loaded with Fluo-4AM (Molecular probes) for 1 hour at room temperature in culture medium (DMEM) supplemented with 10% FCS and 2.5 mM probenecid (Sigma Chemical Co, Poole, Dorset, UK). Cells were then washed with Hanks' balanced saline solution containing 10 mM Hepes, 2.5 mM probenecid, 0.1 mM CaCl_2 _and 1 mM MgCl_2_. The fluorescence was continuously recorded at wavelengths of 485 nm excitation and 520 nm emission using a Flexstation (Molecular Devices, Wokingham, UK). Cells were treated with 10 μM cyclopiazonic acid (final concentration) for 4 minutes followed by the addition of 1.9 mM CaCl_2 _(2 mM final concentration). For agonist induced Ca^2+ ^responses, cells were stimulated with bradykinin (1 μM) or histamine (100 μM final concentration) for 4 minutes in 0.1 mM CaCl_2 _buffer followed by the addition of 1.9 mM CaCl_2_. Data are presented as changes in fluorescence intensity (FI) compared with the baseline, the area under the curve was used as an estimation of changes in [Ca^2+^]_i_.

### Patch-clamp electrophysiology

The conventional whole-cell patch-clamp technique [11] was employed to record store operated inward currents in single HASM cells with an EPC-10 double amplifier and Patchmaster version 2.10 software (HEKA, Lambrecht, Germany). The compositions of the internal and external solutions are as follows; Standard Internal Solution; 110 mM Cs-methanesulfonate, 25 mM CsCl, 2 mM MgCl_2_, 10 mM EGTA, 30 mM HEPES, 3.62 mM CaCl_2_. External Solution: 140 mM NaCl, 5 mM CsCl, 1 mM MgCl_2_, 10 mM D-Glucose, 10 mM HEPES, CaCl_2 _(as indicated). K^+ ^was replaced by Cs^+ ^in both external and internal solutions to block K^+ ^currents and Cl^- ^was replaced by an equal molar concentration of methanesulfonate to minimize Cl^- ^currents. Nifedipine (5 μM) was included in the external solution. Pipettes were drawn from borosilicate glass and had resistances of 5–8 MΩ when filled with internal solution. HASM cells were placed directly into the cell chamber, allowed to settle and then were continuously perfused with external solution at a constant speed of 6 ml/min. Experimental drugs were delivered through a puffer pipette positioned 50 μm around the cells. Cells were held at a membrane potential of -60 mV and current-voltage relationships were analysed every 5s from voltage ramps from -100 to +100 mV at a rate of 0.5 Vs^-1^. Currents were filtered at 1 kHz and sampled at 4 KHz. Individual cell current densities were calculated by dividing peak current amplitude at maximum activation of inward current (at -100 mV) by cell capacitance.

### Immunostaining

HASMs grown on coverslips, transfected with either 20 nM scrambled siRNA or 20 nM STIM1 siRNA were fixed with 4% formaldehyde. Cells were permeabilized (0.5% TritonX-100) and blocked with 20% goat serum in PBS for 20 min. Cells were incubated with primary antibody (mouse, anti-human STIM1 mAb (1:100) (BD Biosciences, Pharmingen) overnight at 4°C followed by labeling with Alexa fluor 488 (Molecular probes). Cells were visualized on a Zeiss LS 510 confocal microscope (Hertfordshire, UK).

### Statistical analysis

Averaged data are presented as mean ± sem. Where appropriate, statistical significance was assessed by unpaired Students T tests or one-way ANOVA followed by the Dunnets test for multiple group comparisons. Data were considered significant at *P < 0.05 or **P < 0.01.

## Results and discussion

**Figure 1:**
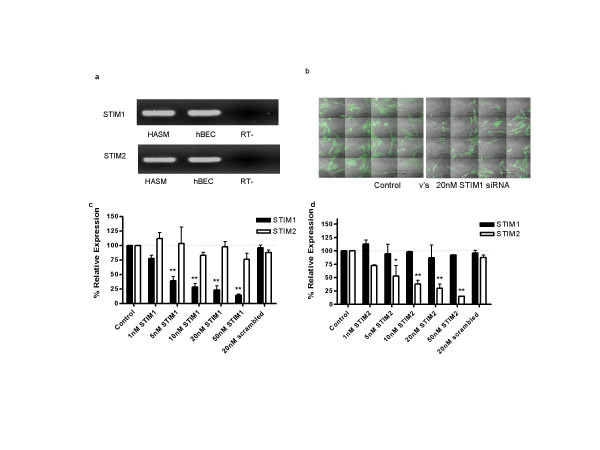
Expression and siRNA mediated knockdown of STIM. **(a) **Expression of STIM1 and STIM2 in human airway myocytes and cultured human bronchial epithelial cells (hbec) using RT-PCR. PCR products were sequenced to confirm expression. **(b) **Tiled arrays of immunofluorescent staining of STIM-1 in HASM cells treated with either 20 nM scrambled siRNA (left) or 20 nM siRNA targeted at STIM1 (right). The intensity of STIM1 staining was decreased in cells treated with STIM1 siRNA. **(c) **siRNA targeted knockdown of STIM1 mRNA assessed by quantitative PCR. Cells transfected with STIM-1 siRNA dose dependently reduced STIM1 mRNA levels (76.9 ± 7.5% at 20 nM) without affecting STIM2 mRNA. **(d) **siRNA targeted knockdown of STIM2 mRNA. Cells transfected with STIM2 siRNA reduced STIM2 mRNA levels (70 ± 8.3% at 20 nM) without affecting STIM1. In addition, transfection of 20 nM scrambled, non-silencing siRNA had no effect on STIM expression.

Initially we assessed the expression of the two known human homologues STIM1 and STIM2 in cells relevant to airway function (figure 1a). Both STIM1 and STIM2 are highly expressed in primary cultures of airway myocytes and also in bronchial epithelial cells. We therefore utilised specific siRNA sequences to target and selectively suppress both STIM1 and STIM2 in order to investigate the role of these putative signals in SOC activation in airway myoctyes. siRNAs targeted at STIM1 and STIM2 produced dose dependent inhibition of the relevant target molecule without significant effects upon expression of either the other STIM homologue or 18sRNA (figure 1c &1d). Transfection of 20 nM siRNA specific for STIM1 or 2 resulted in robust decreases (>70%) of the relevant STIM mRNA. The ability to inhibit protein expression was evaluated by confocal microscopy for STIM1 alone (in view of functional data shown below) with marked inhibition of protein expression being evident following 48 hours exposure to the relevant siRNA (figure 1b).

**Figure 2:**
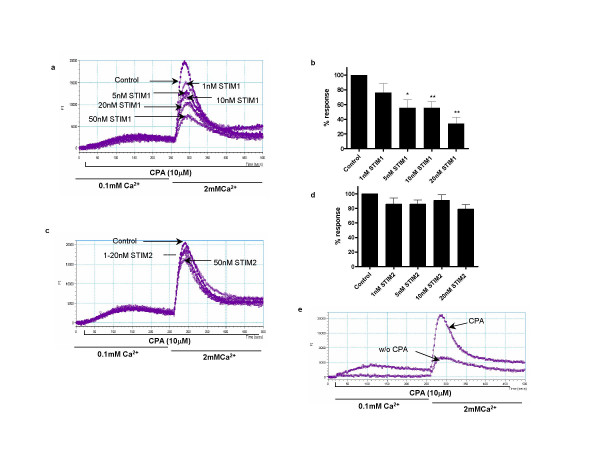
Suppression of STIM1 results in decreased store operated calcium influx. **(a) **A representative raw trace illustrating the changes in [Ca^2+^]_i _(presented as fluorescence intensity (FI)) in HASMs treated with STIM-1 siRNA. CPA (10 μM) was added to the cells in the presence of low extracellular Ca^2+ ^(0.1 mM) followed by the restoration of 2 mM Ca^2+ ^as indicated. **(b) **Summary of the data illustrated in (a) showing averaged changes in fluorescence after 2 mM Ca^2+ ^restoration. **(c) **A representative trace illustrating changes in [Ca^2+^]_i _in HASMs treated with STIM-2 siRNA following the same protocol. **(d) **Summary of the data illustrated in (c) showing averaged changes in fluorescence after 2 mM Ca^2+ ^restoration. The summarized data (c & d) are shown following subtraction of CPA independent (basal) calcium influx (measured as the fluorescence change upon addition of 2 mM Ca^2+ ^to cells not pre-treated with CPA). **(e) **An experimental trace illustrating CPA independent (basal) Ca^2+ ^influx. Results are expressed as % changes ± sem compared to control and represent averaged data from 4 separate experiments. Data are indicated as statistically significant with *P < 0.05 and **P < 0.01.

We next evaluated the ability of siRNA targeted to STIM1 and STIM2 to inhibit SOC using a fluorescence assay utilising Fluo-4AM, designed to measure changes in intracellular free calcium concentration ([Ca^2+^]_i_) in monolayers of cultured human airway myocytes. SOC mediated Ca^2+ ^influx was induced by depletion of the sarcoplasmic reticulum Ca^2+ ^store using a combination of low external Ca^2+^(0.1 mM) and incubation with the SERCA inhibitor cyclopiazonic acid (CPA, 10 μM). Incubation of airway myocytes in low Ca^2+ ^in the presence of CPA resulted in an initial small rise in cytosolic Ca^2+ ^indicative of store depletion. Re-addition of extracellular Ca^2+ ^resulted in rapid influx into the cell through SOCs. siRNA targeted at STIM1 resulted in a dose-dependent reduction of SOC associated Ca^2+ ^influx (figure 2a &2b). Using 20 nM STIM1 siRNA, CPA dependant Ca^2+ ^influx was reduced by 60% compared to control cells. In contrast siRNA targeted at STIM2 had little effect upon SOC activation following store depletion (figure 2c &2d). Control cells initiated both CPA dependent and independent Ca^2+ ^influx (figure 2e) upon Ca^2+ ^re-addition. The mechanism of this basal Ca^2+ ^entry is unknown but previous studies in other cell types suggest that this passive Ca^2+ ^leak may not be due to SOC mediated influx [5]. In keeping with this, CPA independent/basal Ca^2+ ^influx was insensitive to siRNA mediated STIM1 suppression (data not shown).

**Figure 3:**
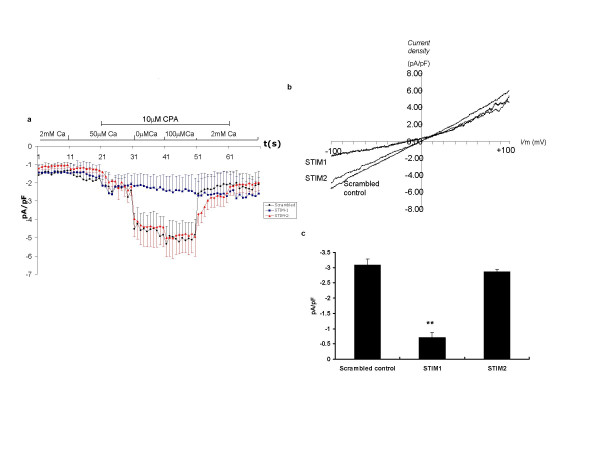
Reduced SOC activated inward current in STIM1 suppressed cells compared with control and STIM2 suppressed cells assessed by whole cell patch clamp. **(a) **A time course of current density (measured at -100 mV), each point represents mean data ± sem of all cells in each group; control cells treated with scrambled siRNA (black circles, n = 16), STIM1 suppressed cells (blue squares, n = 10) and STIM2 suppressed cells (red triangles, n = 12). **(b) **Current-voltage (I-V) relationships at the steady-state SOC current (ie addition of 10 μM CPA in nominally Ca^2+ ^free buffer) and represents averaged data from all cells of each experimental group. **(c) **A bar chart illustrating peak CPA sensitive current density (measured at -100 mV) of cells treated with scrambled control, STIM-1 or STIM-2 siRNA. Data are indicated as statistically significant with **P < 0.01.

We next used whole cell patch clamp electrophysiology approaches to confirm that siRNA targeted at STIM1 but not STIM2 is able to inhibit SOC activation. SOC currents were activated by reducing external Ca^2+ ^and application of 10 μM CPA. Current density was calculated by dividing peak current amplitude by cell capacitance; the average capacitance of the cells was 50.7 ± 4.1 pF (mean ± sem). SOC currents were induced by voltage ramps at a rate of 0.5 mV·ms^-1 ^from a holding potential of -60 mV. Inward currents (SOC currents) measured at -100 mV were then compared. Changes in current density of SOC are illustrated in Figure 3(a–c): preincubation with siRNA targeted to STIM1 almost completely abrogated SOC currents whereas siRNA targeted at STIM2 had no significant effect on SOC activation in these cells. Figure 3b shows the averaged current-voltage (I-V) relationships of the steady-state SOC current (ie addition of 10 μM CPA in Ca^2+ ^free buffer). The STIM1 suppressed cells showed a reduced SOC inward current compared with cells treated with scrambled or STIM2 siRNA sequences. We also noticed a change in the reversal potential in the STIM1 suppressed cells towards a more negative potential: the significance of this is uncertain but similar findings on I_CRAC _currents have been reported using EF-hand mutants of STIM1 in RBL cells [12].

**Figure 4:**
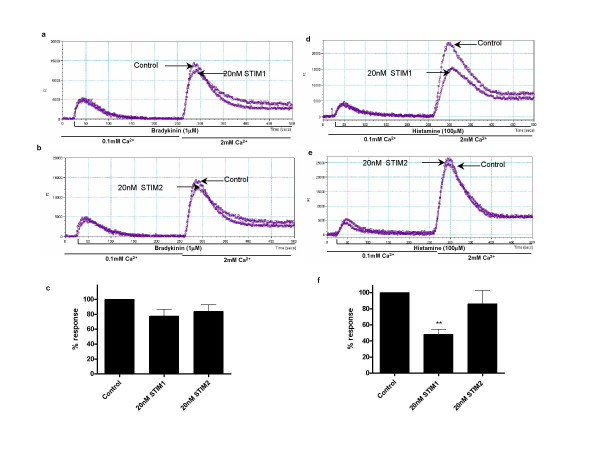
Suppression of STIM1 results in reduced Ca^2+ ^influx in response to histamine but not bradykinin. **(a & b) **Representative traces illustrating bradykinin induced [Ca^2+^]_i _changes in HASMs treated with 20 nM STIM1 siRNA (a) and 20 nM STIM2 siRNA (b) compared with control. **(c) **Summary of data represented in (a & b) showing average changes in fluorescence after 2 mM Ca^2+ ^re-addition. **(d & e) **Representative traces illustrating histamine induced changes in [Ca^2+^]_i _in HASMs treated with 20 nM STIM1 siRNA (d) and 20 nM STIM2 siRNA (e) compared with control. **(f) **Summarized data from (d and e) showing average fluorescence changes after 2 mM Ca^2+ ^re-addition. Bradykinin (1 μM) or Histamine (100 μM) was added to cells in low extracellular Ca^2+ ^(0.1 mM) followed by the restoration of 2 mM Ca^2+ ^as indicated. The summarized data (c & f) are shown with subtraction of agonist independent (basal) calcium influx. Results are expressed as % changes ± sem compared to control and represent averaged data from at least 3 experiments. Data are indicated as statistically significant with *P < 0.05 and **P < 0.01.

The consequences of inhibiting STIM1 and STIM2 expression on agonist mediated Ca^2+ ^entry in human airway myocytes are shown in figure 4. We have previously shown histamine induced Ca^2+ ^responses to be H1 receptor mediated in these cells. Under conditions of low extracellular Ca^2+ ^the sustained rise in intracellular Ca^2+ ^seen following agonist stimulation is reduced, an effect mimicked by a range of di and tri-valent cations including Ni^2+^, La^3+ ^and Gd^3+ ^and also by the putative ROC inhibitor SKF96365 [2]. Previously however it has been impossible to disassociate the SOC and ROC components of the agonist induced Ca^2+ ^response to agonist. Following pre-incubation with siRNA targeted at STIM1 we observed a significant reduction in the magnitude of Ca^2+ ^influx induced by histamine (figure 4d &4f). These results suggest that under the conditions tested the STIM1 mediated SOC component may account for >50% of the Ca^2+ ^influx induced by histamine. We have previously shown histamine to induce a robust activation of phospholipase C in these cells which initiates IP_3 _mediated Ca^2+ ^release from intracellular stores [8]. However there is evidence of agonist specific differences in the ability to activate SOC and ROC in airway myocytes: the ability of siRNA targeted against STIM1 to inhibit bradykinin induced Ca^2+ ^influx was limited (figure 4a and 4c) and markedly less than inhibition seen when histamine was used as the agonist. This may suggest that stimulation of cognate receptors by these agonists vary in their ability to activate SOC or ROC (figure 4). The other possibility is that the difference may reflect variation in the extent of phospholipase C activation: the maximum inositol phospholipid hydrolysis response to bradykinin seen in these cells is around 3 times greater than the maximal response to histamine [8].

The mechanism whereby STIM1 is able to activate SOC in airway myocytes remains to be determined. The STIM genes encode type 1 transmembrane proteins that can potentially form hetero or homo-oligomers via coiled- coiled interactions [13,14]. The NH_2 _terminus contains an EF-hand Ca^2+ ^binding motif which is thought to be responsible for the detection of Ca^2+ ^depletion in stores [6,7,12]. STIM1 is expressed in both plasma and intracellular membranes [13] and the EF hand is thought to be located outside the cell or in the lumen of intracellular stores. It is conceivable that STIM proteins may interact (possibly through coiled coil domains) between the two membranes providing the vital link between intracellular stores and the plasma membrane [5]. Other models have been suggested including translocation of STIM1 from the endoplasmic reticulum to the plasma membrane [7] where STIM1 could directly activate SOC channels, or the involvement of STIM1 in the production of an unidentified Ca^2+ ^influx factor [15].

The exact molecular identity of SOC in airway myocytes remains to be determined although potential candidates include a range of TRP homologues [3,16,17]. At present there are no specific tools to inhibit these channels directly and such approaches may be complicated by the formation of channels formed of heteromeric subunits.

## Conclusion

Our data clearly implicates a role for STIM1 in SOC activation in airway myocytes providing for the first time molecular insight into this key signalling pathway in smooth muscle. Given the importance of control of intracellular Ca^2+ ^to airway smooth muscle contraction STIM1 may provide a potential therapeutic target for diseases characterised by increased smooth muscle contractility such as asthma. However, one note of caution must be added in that STIM1 was initially identified as a candidate tumour suppressor gene [18] and the consequences therefore of long term inhibition of STIM1 expression need to be explored further.

## Competing interests

The author(s) declare that they have no competing interests.

## Authors' contributions

Samantha Peel performed the mRNA expression and calcium studies, Bo Liu performed the electrophysiology and Ian P Hall wrote the paper. All authors were involved in the design of the studies, discussion of the results and preparation of the final manuscript.
